# Nutritional Indices as Non-Invasive Biomarkers: PNI and CONUT Scores Predict Clinical and Endoscopic Activity in Ulcerative Colitis

**DOI:** 10.3390/nu18050829

**Published:** 2026-03-04

**Authors:** Ozgur Yildirim, Mehmet Bayram, Ali Riza Koksal, Hafize Uzun, Omur Tabak

**Affiliations:** 1 Department of Internal Medicine, Kanuni Sultan Suleyman Training and Research Hospital, University of Health Sciences, 34303 Istanbul, Türkiye; drozguryildirim@outlook.com; 2 Department of Gastroenterology, Türkiye Hospital, 34381 Istanbul, Türkiye; drmhbayram@gmail.com; 3 Department of Internal Medicine, Billings Clinic, Billings, MT 59101, USA; arkoksal@gmail.com; 4 Department of Medical Biochemistry, Faculty of Medicine, Istanbul Atlas University, 34403 Istanbul, Türkiye; huzun59@hotmail.com

**Keywords:** ulcerative colitis, prognostic nutritional index, controlling nutritional status score, Mayo score, Rachmilewitz index

## Abstract

***Background and Objectives***: Determination of disease activity is important for appropriate treatment selection and clinical follow-up in ulcerative colitis (UC). Colonoscopic examination is the gold standard in the evaluation of disease activity in current clinical practice. However, colonoscopic procedures are invasive, expensive, and not comfortable for the patients; also, possible complications that may develop during the procedure have led clinicians to seek practical, economical, and reproducible new biomarkers that can be used in the clinical follow-up of UC patients. In our study, we aimed to analyze the relationship between the nutritional markers, such as prognostic nutritional index (PNI) and controlling nutritional status score (CONUT), with clinical and endoscopic activities of the disease in ulcerative colitis disease. ***Materials and Methods***: The study included 196 patients who were followed up with the diagnosis of ulcerative colitis in the Internal Medicine Clinic and Gastroenterology Outpatient Clinic of our hospital. Demographic data, laboratory parameters, and endoscopy reports of the patients were reviewed retrospectively. Patients were divided into two groups: remission-mild disease and moderate-severe disease in terms of clinical activity, and endoscopic remission and active disease in terms of endoscopic activity. The relationship between the PNI and CONUT scores calculated from the results of the examinations at the hospital admissions with the Mayo score and Rachmilewitz endoscopic activity index scores stated in the endoscopy reports was analyzed. ***Results***: In our study, 78 (39.8%) cases were female and 118 (60.2%) were male. The mean age was 40.28 ± 13.74 years. According to clinical activity, 90 (45.9%) of the cases had remission-mild disease, and 106 (54.1%) had moderate-to-severe disease. According to endoscopic activity, endoscopic remission was observed in 66 (33.7%) cases, and active disease was observed in 130 (66.3%) cases. PNI score was detected to be statistically significantly lower in the moderate-to-severe clinical activity group and endoscopically active patient group compared to the other groups, while the CONUT score was detected to be statistically significantly higher (*p* < 0.001; *p* < 0.001). ***Conclusions***: PNI and CONUT scores are not only effective predictors of clinical and endoscopic disease activity in UC but also emphasize the critical role of nutritional assessment in the holistic management of inflammatory bowel disease. These findings suggest that integrating routine nutritional screening into gastroenterology practice can provide dual benefits: serving as a non-invasive biomarker for mucosal inflammation and identifying patients who may require early nutritional intervention to improve clinical outcomes.

## 1. Introduction

Ulcerative Colitis (UC) is an idiopathic and chronic inflammatory disease that affects the colon, starting from the rectum and extending to the proximal colon segments [1]. Inflammation is characteristically limited to the mucosal surface, starting in the rectum and usually extending proximally throughout the colon [2]. The disease is classified as proctitis, left-sided colitis, and extensive colitis (pancolitis) according to the degree of colon involvement [3].

In UC, patients present clinically with abdominal pain, diarrhea, and hematochezia, while symptoms may also be accompanied by tenesmus and incontinence [4]. In the treatment of the disease, which progresses with periods of attack and remission, mesalazine and, when necessary, corticosteroids, immunosuppressive agents, and biological agents are preferred [5]. The aim of treatment is to induce and maintain remission. Accurate determination of the severity of clinical activity in the disease by the clinician during follow-up of the disease with remissions and relapses is important for the selection of appropriate treatment and prediction of prognosis [6].

Classifications and scoring systems such as the Rachmilewitz Endoscopic Activity Index (Table 1) and the Mayo Score (Table 2), which are created using laboratory, clinical, and endoscopic findings, or a combination of these, to determine the severity of disease activity, assist clinical practice in predicting prognosis in patients and determining appropriate treatment options [7].

Endoscopic examination has an important place in the diagnosis of the disease, detection and monitoring of its activity, and selection of appropriate agents for treatment [8]. In addition to all this, the fact that the procedure is expensive, uncomfortable for the patient, and carries significant complication risks, such as perforation that may occur during the procedure, has led clinicians to search for new non-invasive biomarkers that are cheaper, more practical, and more effective [9].

When the literature is reviewed, nutritional markers such as prognostic nutritional index (PNI) and controlling nutritional status score (CONUT) have been used to predict disease activity and prognosis in different diseases [10,11]. PNI is calculated with the formula: 10 × albumin (g/dL) + 0.005 × lymphocytes (/mm^3^) [12]. It has been shown that the CONUT score, which is generated by utilizing lymphocyte, albumin, and cholesterol levels (Table 3), can be used to evaluate the nutritional level of patients [13].

In our study, we examined the demographic data, laboratory data, endoscopy reports, nutritional scores (PNI and CONUT), Rachmilewitz Endoscopic Activity Index, and Mayo Score/Disease Activity Index scores of patients followed up with a diagnosis of UC in our hospital’s internal medicine and gastroenterology clinics. It was aimed to investigate the predictability of clinical and endoscopic activity in patients by non-invasive methods via these nutritional scores.

## 2. Methods

A retrospective evaluation was made of 196 patients aged 18 years and over with UC, who were followed up at the Internal Medicine and Gastroenterology Clinics of Istanbul Kanuni Sultan Süleyman Training and Research Hospital between 2018 and 2023, and whose retrospective patient files and laboratory results yielded sufficient data. All data used within the scope of the study were obtained from the hospital automation system by a retrospective file scanning method.

Patients’ age, gender, complete blood count parameters, CRP, albumin, total cholesterol values, Mayo Clinic Activity Index scores, and Rachmilewitz endoscopic activity index scores in endoscopy reports were recorded. PNI and CONUT scores were calculated with the data obtained. According to the Mayo score, patients were divided into two groups in terms of clinical activity: remission-mild disease (MAYO < 6) and moderate-severe disease (MAYO ≥ 6). According to the Rachmilewitz score, patients were divided into two groups in terms of endoscopic activity: active disease (Score ≥ 4) and remission group (Score < 4). It was investigated whether there is a relationship between PNI and CONUT scores and clinical and endoscopic disease activity of patients.

The exclusion criteria were as follows: patients under 18 years of age; individuals diagnosed with solid organ malignancies, leukemia, or lymphoma; those with liver cirrhosis or chronic renal failure (including nephrotic-level proteinuria, diabetic and hypertensive nephropathy); and cachectic patients or those with a history of cerebrovascular accidents likely to present with hypoalbuminemia. Additionally, patients with hyperlipidemia (triglycerides > 250 mg/dL) or those receiving antihyperlipidemic therapy were excluded to avoid interference with the lipid-based components of the nutritional scores.

## 3. Statistical Analysis

SPSS version 23.0 for Windows (IBM Corporation, Chicago, IL, USA) was used for statistical analyses. The variables were investigated using visual (histograms) and analytical methods (Kolmogorov–Smirnov/Shapiro–Wilk tests) to determine whether or not they were normally distributed. The Chi-square test was used for comparisons of categorical variables. Continuous variables were shown as mean ± standard deviation or median (interquartile range) according to their distribution pattern. Ordinal variables and continuous variables that do not have a normal distribution were compared by the Mann–Whitney U test. Student’s *t*-test was used to evaluate differences between the two groups in normally distributed continuous variables. Pearson correlation analysis was performed for variables with normal distribution, and Spearman correlation analysis was performed for variables that do not have distribution. To find optimal cutoffs, individual receiver operating characteristic curve (ROC) analysis was performed for the PNI and CONUT scores. A value of *p* < 0.05 (two-sided) was considered statistically significant.

## 4. Results

In this study, data of 196 patients aged between 19 and 70 were analyzed; 78 (39.8%) of the patients were female, and 118 (60.2%) were male. The mean age of the patients was 40.28 ± 13.74. According to clinical disease activity, a total of 90 (45.9%) of the patients were in remission and had mild disease, while 106 (54.1%) had moderate and severe disease. When endoscopic disease activity was analyzed, 33.7% of the patients were in endoscopic remission, while 66.3% had endoscopically active disease.

When the patients were examined according to Mayo clinic activity groups, a statistically significant difference was detected between the PNI and CONUT scores of the moderate-severe disease activity group and the remission-mild disease activity groups (*p* < 0.001, *p* < 0.001) (Table 4). There was also a statistically significant difference between the two groups mentioned in terms of hemoglobin level, platelet count, lymphocyte count, CRP, sedimentation, albumin, and total cholesterol levels (*p* < 0.05 for all). However, no statistically significant difference was found between age, MPV, and RDW levels (*p* > 0.05 for all).

Patients were also analyzed according to Rachmilewitz endoscopic activity groups, and a statistically significant difference was found between the PNI and CONUT scores of the endoscopically active disease group and the remission groups (*p* < 0.001, *p* < 0.001) (Table 5). There was a statistically significant difference between the groups in terms of hemoglobin level, platelet count, lymphocyte count, CRP, sedimentation, albumin, and total cholesterol levels (*p* < 0.05 for all). There was no statistically significant difference between the two groups for age, MPV, and RDW levels (*p* > 0.05 for all).

In our study, ROC analysis was performed, and ROC curves were drawn to evaluate the relationship between the PNI and CONUT scores of the patients and the moderate-to-severe clinical disease activity according to the Mayo score and the presence of endoscopically active disease according to the Rachmilewitz score.

**Figure 1:**
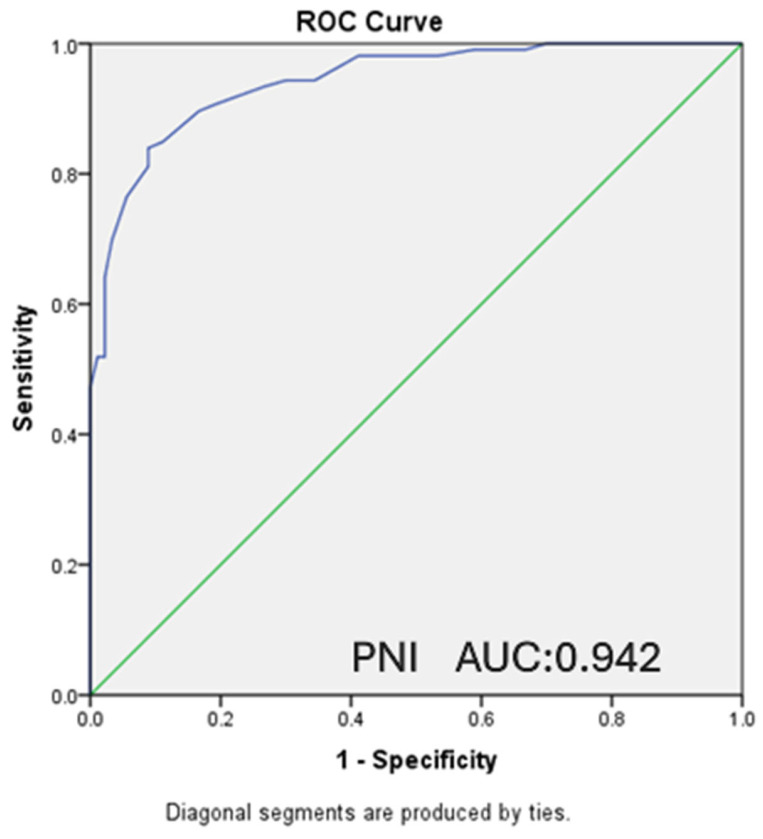
ROC curve presenting the relationship between PNI and moderate-severe clinical activity. (AUC: 0.942 CI: 0.912–0.972 *p* < 0.001) (PNI ≤ 50.25 sensitivity: 84%, specificity: 91.1%).

**Figure 2:**
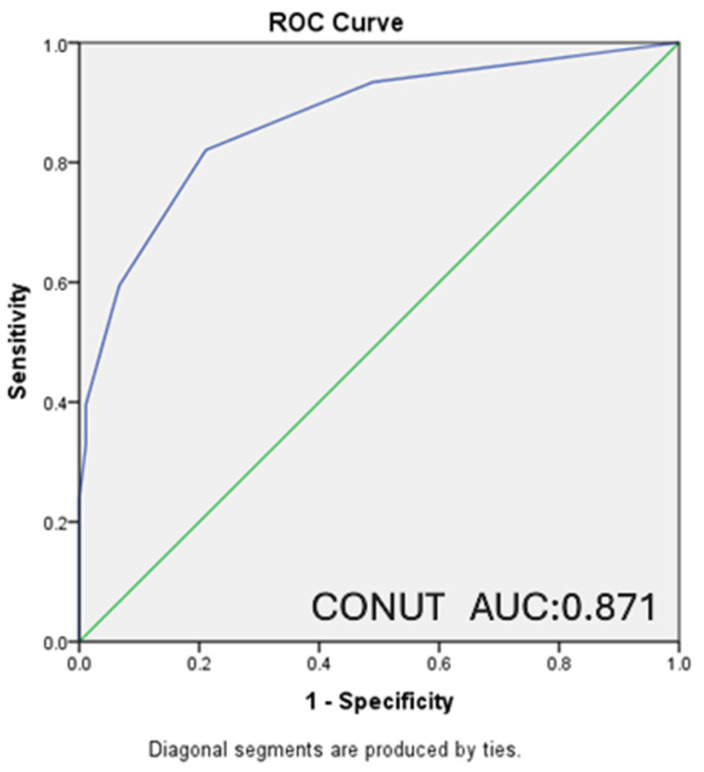
ROC curve presenting the relationship between CONUT score and moderate-severe clinical activity. (AUC: 0.871 CI: 0.822–0.920 *p* < 0.001) (CONUT ≥ 2 sensitivity: 82.1%, specificity: 78.9%).

A statistically significant inverse relationship was found between PNI score and moderate–severe clinical disease activity, with lower PNI values observed in patients with higher disease severity (AUC: 0.942, CI: 0.912–0.972, *p* < 0.001). For PNI ≤ 50.25, which was determined as the cut-off value, sensitivity was 84%, and specificity was 91.1%. A statistically significant relationship was also detected between CONUT score and moderate-severe clinical activity (AUC: 0.871, CI: 0.822–0.920, *p* < 0.001). For CONUT ≥ 2, which was determined as the cut-off value, sensitivity was calculated as 82.1% and specificity as 78.9% (Figure 1 and Figure 2).

**Figure 3:**
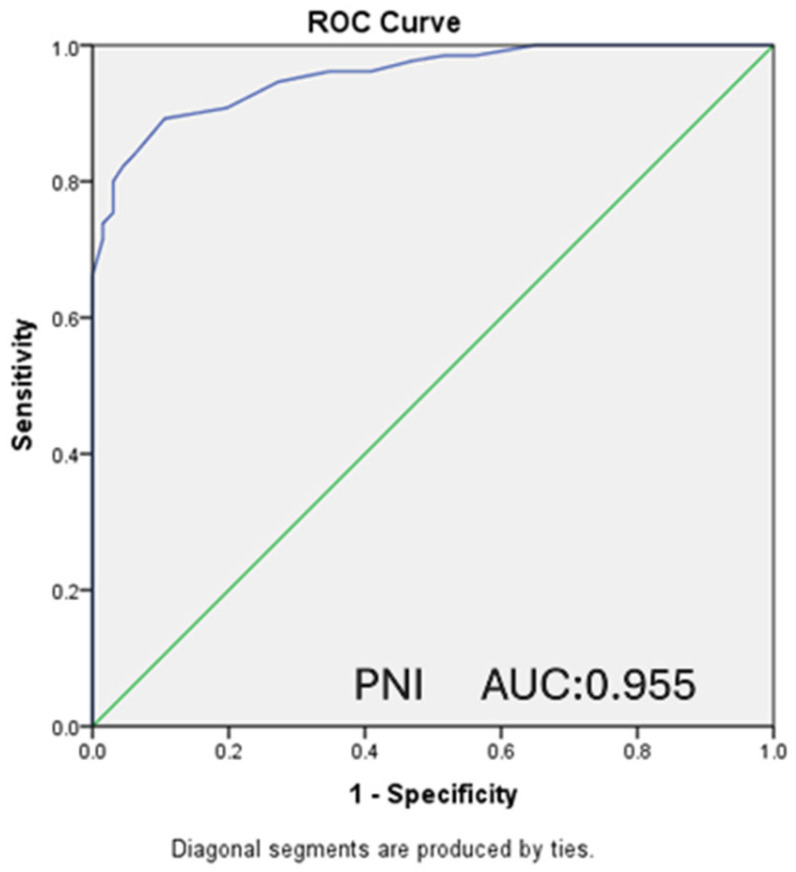
ROC curve presenting the relationship between PNI and endoscopic activity. (AUC: 0.955 CI: 0.930–0.980 *p* < 0.001) (PNI ≤ 52.75 sensitivity: 89.2% specificity: 89.4%).

**Figure 4:**
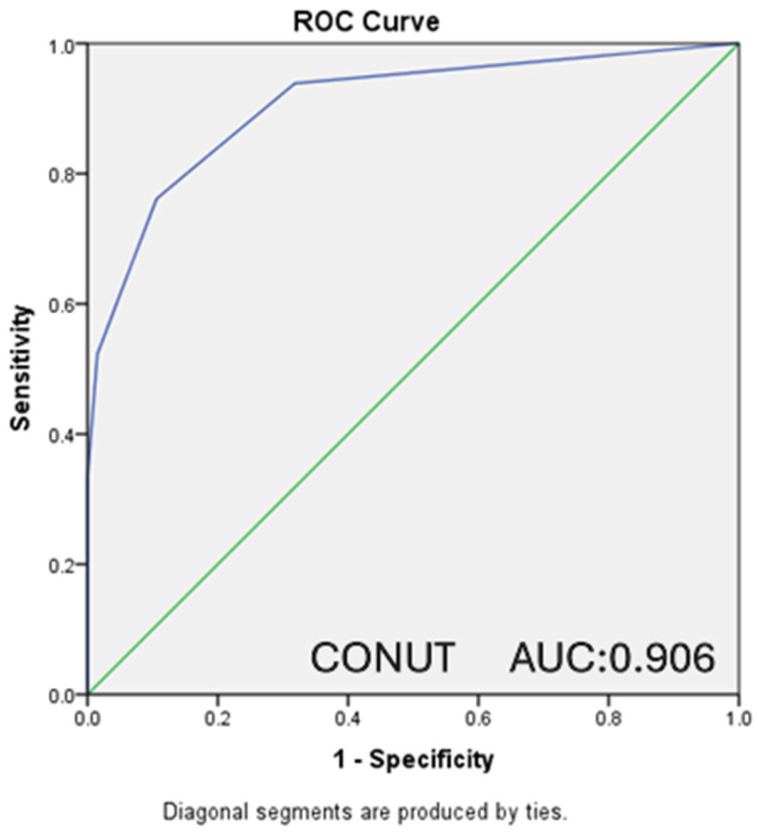
ROC curve presenting the relationship between CONUT score and endoscopic activity. (AUC: 0.906 CI: 0.864–0.948 *p* < 0.001) (CONUT ≥ 2 sensitivity: 76.2%, specificity: 89.4%).

A statistically significant relationship was detected in the ROC analysis between the presence of endoscopically active disease and PNI scores (AUC: 0.955, CI: 0.930–0.980, *p* < 0.001). For PNI ≤ 52.75, which was determined as the cut-off value, sensitivity was 89.2%, and specificity was 89.4% (Figure 3). A statistically significant relationship was also detected in the ROC analysis between the presence of endoscopically active disease and the CONUT score (AUC: 0.906, CI: 0.864–0.948, *p* < 0.001). For CONUT ≥ 2, which was determined as the cut-off value, sensitivity was calculated as 76.2% and specificity as 89.4% (Figure 4).

## 5. Discussion

Endoscopic examination is the gold standard for diagnosing UC and determining its activity, as well as for evaluating the treatment response in the clinical course. However, the invasive nature of the procedure, its cost, its discomfort for the patient, possible complications, and contraindications have led clinicians to search for new non-invasive markers that can be used in patient follow-up. Emergence of new non-invasive biomarkers that are practical, economical, easily accessible, reproducible, and also provide rapid results will significantly facilitate physicians’ patient follow-up in clinical practice. In our study, we intended to evaluate the usability of PNI and CONUT scores as non-invasive markers in predicting the clinical and endoscopic activity of the disease.

The prognostic nutritional index, first described by Onodera et al., was developed to examine malnutrition and associate surgical risks in patients with malignancy, and revealed that low PNI scores had increased surgical risk [12]. The index, which is gaining popularity academically with different clinical studies today, has been investigated for its predictability of disease prognosis in a wide clinical spectrum from different malignancy groups to patients with heart failure and diabetic nephropathy [14,15,16].

When we review the studies conducted on PNI levels of patients diagnosed with UC in the literature, studies that evaluate PNI levels in terms of post-operative complications and mortality in patients who underwent surgery due to UC stand out. In the study conducted by Chohno et al., evaluating 1151 patients retrospectively who underwent surgery for UC, a significant relationship was found between lower PNI scores and surgical mortality [17]. Also, in the study conducted by Kato et al., including 140 patients who underwent surgery for UC, a significant relationship was found between the risk of developing postoperative complications and lower PNI levels in UC patients [18]. In another study conducted by Okita et al., including 110 patients who underwent surgery for UC, it was stated that the risk of infectious complications in the postoperative period was significantly increased in patients with PNI <47 [19]. Although our study differs from other studies by examining the clinical and endoscopic activity of the disease instead of postoperative complications, lower PNI levels were found to be associated with active disease in our study.

Although there are not many studies in the literature evaluating the CONUT score levels in patients diagnosed with UC, the study conducted by Sobolewska-Włodarczyk et al. stands out. That study investigated the relationship between patients’ CONUT scores and response to 14 weeks of vedolizumab treatment. Patients with lower pre-treatment CONUT scores were significantly more likely to achieve remission after treatment [20]. In our study evaluating clinical and endoscopic activity, similarly, lower CONUT scores were found to be associated with remission, and higher CONUT scores were found to be associated with active disease. Thus, our findings support the clinical utility of PNI and CONUT as non-invasive biomarkers reflecting overall inflammatory burden rather than isolated drivers of endoscopic activity.

When the relationship between disease activity and lymphocyte levels in UC was examined, in the study conducted by Çifci et al., no significant relationship was found between endoscopic activity and lymphocyte count; however, in the study conducted by Cui et al., lymphocyte levels were found to be significantly lower in endoscopically active disease [21,22]. We concluded that lymphocyte levels were significantly lower in clinically and endoscopically active disease compared to remission groups in our study, similar to the study by Cui et al. This condition can be evaluated as secondary to malnutrition and increased autoimmunity caused by active disease in patients.

Albumin is a negative acute phase reactant, and hypoalbuminemia may be seen in inflammatory conditions. Hypoalbuminemia in UC may be secondary to increased albumin catabolism caused by active inflammation, malnutrition, and intestinal malabsorption [23]. Lower albumin levels have been considered among the poor prognostic factors in predicting the disease clinic by the American College of Gastroenterology (ACG) [24]. In our study, supporting the literature, albumin levels were found to be statistically significantly lower in clinically and endoscopically active patients compared to the remission group.

Hypocholesterolemia has been recognized as one of the strong indicators of malnutrition [25]. Hypocholesterolemia may be observed in patients with UC as a result of malabsorption caused by the disease and the resulting malnutrition. In fact, in our study, it was observed that total cholesterol levels were statistically significantly lower in endoscopically and clinically active patients compared to patients in remission.

PNI is calculated using lymphocyte count and albumin, and the CONUT score is calculated using lymphocyte count, albumin, and total cholesterol levels. In our study, each subparameter constituting the PNI and CONUT scores was significantly associated with clinical and endoscopic activity on its own, which led to a very strong and significant relationship between the PNI and CONUT scores that used these parameters and disease activity. Since PNI and CONUT scores have considerably high sensitivity and specificity in terms of clinical and endoscopic disease activity, these scores can be considered promising in terms of their usability in clinical practice in the future.

Despite the clinical utility of PNI and CONUT scores, several factors must be considered during interpretation. Albumin and cholesterol are not only nutritional markers, but also negative acute-phase reactants influenced by the severity of systemic inflammation. Therefore, while these scores provide valuable prognostic information, they cannot be used interchangeably with more specific markers such as fecal calprotectin or C-reactive protein. Furthermore, the increasing prevalence of obesity in UC (ranging from 20% to 40% in various cohorts) presents a challenge; patients with sarcopenic obesity or metabolically abnormal obesity may present with misleadingly stable indices despite underlying nutritional deficits. Additionally, the influence of corticosteroids and biological therapies on lymphocyte counts and lipid profiles should be acknowledged as potential confounders in the clinical application of these scores.

As well as the positive results obtained, our study also has some limitations. The main ones are that our study was a retrospective study and included single-center data. Being a single-center study also precluded the study from reaching higher case numbers. In addition, the fact that only the Mayo Clinic Activity and Rachmilewitz Endoscopic Activity Index results of the patients were available through endoscopy reports and that the patients could not be divided into additional groups according to the Truelove-Witts and Montreal classifications that can be used in UC clinical practice may be considered as another limitation of the study. In the future, as a result of multicenter studies with higher patient numbers and including different additional clinical and endoscopic activity indices, the importance of the parameters in clinical practice may be further strengthened by having more and clearer information about the relationship between our relevant parameters and disease activity.

## 6. Conclusions

This study demonstrated a significant correlation between PNI and CONUT scores and both clinical and endoscopic disease activity in patients with UC. Our findings suggest that PNI and CONUT scores are not only valuable non-invasive markers for predicting disease severity and mucosal inflammation, but also essential tools for identifying the nutritional deterioration that accompanies active UC. Integrating these nutritional indices into routine clinical practice can facilitate the early identification of patients at risk of malnutrition, enabling timely nutritional support and more precise monitoring of disease progression. Consequently, assessing nutritional status should be considered an integral part of the clinical and endoscopic evaluation of UC patients to improve overall management and therapeutic outcomes.

## Figures and Tables

**Table 1:** Rachmilewitz Endoscopic Activity Index.

Endoscopic Finding	Score
Mucosal Granulation	
Yes	0
No	2
Vascular pattern	
Normal	0
Disturbed/Faded	1
Absent	2
Mucosal Fragility	
None	0
Slightly increased (bleeding after contact)	2
Greatly increased (bleeding spontaneously)	4
Mucosal damage (ulcer, erosion, exudate, fibrin, mucus)	
None	0
Slight	2
Pronounced	4
Patients with a score of ≥4 are considered endoscopically active.	

**Table 2:** Mayo Score/Clinical Disease Activity Index for Ulcerative Colitis.

Stool frequency	0: Normal
	1: 1–2 stools/day more than normal
	2: 3–4 stools/day more than normal
	3: ≥5 stools/day more than normal
Rectal Bleeding	0: No bleeding
	1: Visible linear bleeding less than half the time
	2: Visible bleeding with stool half of the time and more
	3: Passing blood alone
Mucosal appearance at endoscopy	0: Inactive disease
	1: Mild disease (erythema, decreased vascular pattern, mild friability)
	2: Moderate disease (marked erythema, absent vascular pattern, erosions)
	3: Severe disease (spontaneous bleeding, ulceration)
Physician rating for disease activity	0: Normal
	1: Mild disease
	2: Moderate disease
	3: Severe disease
Total Score	0–2: Remission
	3–5: Mild disease activity
	6–10: Moderate disease activity
	11–12: Severe disease activity

**Table 3:** CONUT Score.

Malnutrition	Normal	Mild	Moderate	Severe
Albumin (g/dL)	≥3.5 (0 point)	3–3.4 (2 points)	2.5–2.9 (4 points)	<2.5 (6 points)
Total Lymphocyte Count (mm^3^)	≥1600 (0 point)	1200–1599 (1 point)	800–1199 (2 points)	<800 (3 points)
Total Cholesterol (mg/dL)	≥180 (0 point)	140–179 (1 point)	100–139 (2 points)	<100 (3 points)
Score	0–1	2–4	5–8	9–12

**Table 4:** Evaluation of parameters affecting the severity of clinical activity in patients.

	Clinical Activity		
	Remission-Mild (n: 90)	Moderate-Severe (n: 106)	*p*
Age	39.5 (31–50.2)	37 (27.7–52.2)	^a^ 0.518
WBC (10^3^/μL)	7.35 (6–8.8)	8.25 (6.7–10.27)	**^a^** **0.006**
Neutrophil (10^3^/μL)	4.25 (3.3–5.52)	5.7 (4–8.02)	**^a^** **<0.001**
HGB (10^3^/μL)	13 (11.7–14.3)	11.8 (9.7–13.4)	**^a^** **<0.001**
PLT (10^3^/μL)	280 (231–345)	322 (237–401)	**^a^** **0.007**
MPV (fL)	10 (9.37–10.92)	9.7 (9–10.62)	^a^ 0.092
RDW (%)	13.4 (12.6–14.75)	13.5 (12.67–16)	^a^ 0.278
CRP (mg/dL)	3 (1.7–6.2)	27.5 (9–67.2)	**^a^** **<0.001**
ESR (mm/h)	14 (7–25)	35 (16–54)	**^a^** **<0.001**
Albumin (g/dL)	4.6 (4.3–4.8)	3.7 (3.1–4.1)	**^a^** **<0.001**
CONUT Score	0 (0–1)	3 (2–5)	**^a^** **<0.001**
Lymphocyte (10^3^/μL)	2.08 ± 0.6	1.58 ± 0.51	**^b^** **<0.001**
Total Cholesterol mg/dL)	184.31 ± 38.68	138.09 ± 39.39	**^b^** **<0.001**
PNI Score	55.6 ± 4.3	43.5 ± 7.4	**^b^** **<0.001**

Values are presented using means ± standard deviations for normally distributed and medians and first and third quartiles in brackets for the non-normally distributed variables. WBC: White blood cell; HGB: Hemoglobin; PLT: Platelet; MPV: Mean platelet volume; RDW: Red blood cell distribution width; CRP: C-reactive protein; ESR: Erythrocyte sedimentation rate. a: Mann–Whitney U test; b: Student’s *t*-test. Bold: means *p* values lower than 0.05.

**Table 5:** Evaluation of parameters affecting endoscopic activity and remission in patients.

	Endoscopical Activity		
	Remission (n: 66)	Active Disease (n: 130)	*p*
Age	40.5 (31–52.5)	38 (28–51)	^a^ 0.394
WBC (10^3^/μL)	7.35 (6–8.8)	8.03 (6.57–8.92)	**^a^** **0.035**
Neutrophil (10^3^/μL)	4.1 (3.3–5.52)	5.3 (3.97–7.55)	**^a^** **<0.001**
HGB (10^3^/μL)	13.1 (11.8–15)	12 (10.17–13.52)	**^a^** **<0.001**
PLT (10^3^/μL)	284 (228–339)	318 (237–385)	**^a^** **0.024**
MPV (fL)	10 (9.2–10.92)	9.8 (9.17–10.7)	^a^ 0.278
RDW (%)	13.4 (12.47–14.9)	13.5 (12.7–15.55)	^a^ 0.277
CRP (mg/dL)	2 (1–5)	18.5 (7.7–55.2)	**^a^** **<0.001**
ESR (mm/h)	12 (6–24)	32 (15–48)	**^a^** **<0.001**
Albumin (g/dL)	4.6 (4.5–4.8)	3.8 (3.27–4.2)	**^a^** **<0.001**
CONUT Score	0 (0–1)	3 (2–5)	**^a^** **<0.001**
Lymphocyte (10^3^/μL)	2.2 ± 0.61	1.61 ± 0.49	**^b^** **<0.001**
Total Cholesterol (mg/dL)	197.1 ± 33.95	140.13 ± 33.66	**^b^** **<0.001**
PNI Score	57.1 ± 3.6	45.1 ± 7.5	**^b^** **<0.001**

Values are presented using means ± standard deviations for normally distributed and medians and first and third quartiles in brackets for the non-normally distributed variables. WBC: White blood cell; HGB: Hemoglobin; PLT: Platelet; MPV: Mean platelet volume; RDW: Red blood cell distribution width; CRP: C-reactive protein; ESR: Erythrocyte sedimentation rate. a: Mann-Whitney U test; b: Student’s *t*-test. Bold: means *p* values lower than 0.05.

## Data Availability

The original contributions presented in this study are included in the article. Further inquiries can be directed to the corresponding author.

## References

[1] Ungaro R., Mehandru S., Allen P.B., Peyrin-Biroulet L., Colombel J.-F. (2017). Ulcerative colitis. Lancet.

[2] Ordás I., Eckmann L., Talamini M., Baumgart D.C., Sandborn W.J. (2012). Ulcerative colitis. Lancet.

[3] Silverberg M.S., Satsangi J., Ahmad T., Arnott I.D., Bernstein C.N., Brant S.R., Caprilli R., Colombel J.-F., Gasche C., Geboes K. (2005). Toward an Integrated Clinical, Molecular and Serological Classification of Inflammatory Bowel Disease: Report of a Working Party of the 2005 Montreal World Congress of Gastroenterology. Can. J. Gastroenterol. Hepatol..

[4] Adams S.M., Bornemann P.H. (2013). Ulcerative colitis. Am. Fam. Physician.

[5] Rogler G. (2014). Chronic ulcerative colitis and colorectal cancer. Cancer Lett..

[6] Pabla B.S., Schwartz D.A. (2020). Assessing Severity of Disease in Patients with Ulcerative Colitis. Gastroenterol. Clin. N. Am..

[7] Cooney R.M., Warren B.F., Altman D.G., Abreu M.T., Travis S.P. (2007). Outcome measurement in clinical trials for ulcerative colitis: Towards standardisation. Trials.

[8] Spiceland C.M., Lodhia N. (2018). Endoscopy in inflammatory bowel disease: Role in diagnosis, management, and treatment. World J. Gastroenterol..

[9] Chen P., Zhou G., Lin J., Li L., Zeng Z., Chen M., Zhang S. (2020). Serum Biomarkers for Inflammatory Bowel Disease. Front. Med..

[10] Shirakabe A., Hata N., Kobayashi N., Okazaki H., Matsushita M., Shibata Y., Nishigoori S., Uchiyama S., Asai K., Shimizu W. (2018). The prognostic impact of malnutrition in patients with severely decompensated acute heart failure, as assessed using the Prognostic Nutritional Index (PNI) and Controlling Nutritional Status (CONUT) score. Heart Vessel..

[11] Akkuzu M.Z., Altıntaş E., Yaraş S., Sezgin O., Ateş F., Üçbilek E., Özdoğan O. (2022). Controlling Nutritional Status (CONUT) Score and Prognostic Nutritional Index (PNI) Are Good Candidates for Prognostic Markers for Acute Pancreatitis. Medicina.

[12] Onodera T., Goseki N., Kosaki G. (1984). Prognostic nutritional index in gastrointestinal surgery of malnourished cancer patients. Nihon Geka Gakkai Zasshi.

[13] Ignacio de Ulíbarri J., González-Madroño A., de Villar N.G., González P., González B., Mancha A., Rodríguez F., Fernández G. (2005). CONUT: A tool for controlling nutritional status. First validation in a hospital population. Nutr. Hosp..

[14] Zhang Q., Bao J., Zhu Z.-Y., Jin M.-X. (2021). Prognostic nutritional index as a prognostic factor in lung cancer patients receiving chemotherapy: A systematic review and meta-analysis. Eur. Rev. Med. Pharmacol. Sci..

[15] Cheng Y., Sung S., Cheng H., Hsu P., Guo C., Yu W., Chen C. (2017). Prognostic Nutritional Index and the Risk of Mortality in Patients with Acute Heart Failure. J. Am. Heart Assoc..

[16] Zhang J., Xiao X., Wu Y., Yang J., Zou Y., Zhao Y., Yang Q., Liu F. (2022). Prognostic Nutritional Index as a Predictor of Diabetic Nephropathy Progression. Nutrients.

[17] Chohno T., Uchino M., Sasaki H., Bando T., Takesue Y., Ikeuchi H. (2018). Associations Between the Prognostic Nutritional Index and Morbidity/Mortality During Intestinal Resection in Patients with Ulcerative Colitis. World J. Surg..

[18] Kato H., Seishima R., Nakamura K., Matsui S., Shigeta K., Okabayashi K., Kitagawa Y. (2023). The Prognostic Nutritional Index is a Predictive Marker for Postoperative Complications in Patients with Late-Onset Ulcerative Colitis. World J. Surg..

[19] Okita Y., Araki T., Okugawa Y., Kondo S., Fujikawa H., Hiro J., Inoue M., Toiyama Y., Ohi M., Uchida K. (2019). The prognostic nutritional index for postoperative infectious complication in patients with ulcerative colitis undergoing proctectomy with ileal pouch-anal anastomosis following subtotal colectomy. J. Anus Rectum Colon.

[20] Sobolewska-Włodarczyk A., Walecka-Kapica E., Włodarczyk M., Gąsiorowska A. (2023). Nutritional Status Indicators as a Predictor of Achieving Remission at Week 14 during Vedolizumab Therapy in Patients with Ulcerative Colitis: A Pilot Study. Nutrients.

[21] Cifci S., Ekmen N. (2021). Prediction of Mucosal Health by NLR, CRP x NLR and MPV in Ulcerative Colitis: Can Their Availability Change According to Treatment Options?. Cureus.

[22] Cui J., Li X., Zhang Z., Gao H., Li J. (2022). Common laboratory blood test immune panel markers are useful for grading ulcerative colitis endoscopic severity. BMC Gastroenterol..

[23] Khan N., Patel D., Shah Y., Trivedi C., Yang Y.-X. (2017). Albumin as a prognostic marker for ulcerative colitis. World J. Gastroenterol..

[24] Rubin D.T., Ananthakrishnan A.N., Siegel C.A., Sauer B.G., Long M.D. (2019). ACG Clinical Guideline: Ulcerative Colitis in Adults. Am. J. Gastroenterol..

[25] Zhang Z., Pereira S.L., Luo M., Matheson E.M. (2017). Evaluation of Blood Biomarkers Associated with Risk of Malnutrition in Older Adults: A Systematic Review and Meta-Analysis. Nutrients.

